# Potential use of molecular and structural characterization of the gut bacterial community for postmortem interval estimation in Sprague Dawley rats

**DOI:** 10.1038/s41598-020-80633-2

**Published:** 2021-01-08

**Authors:** Huan Li, Siruo Zhang, Ruina Liu, Lu Yuan, Di Wu, E. Yang, Han Yang, Shakir Ullah, Hafiz Muhammad Ishaq, Hailong Liu, Zhenyuan Wang, Jiru Xu

**Affiliations:** 1 Department of Microbiology and Immunology, School of Basic Medical Sciences, Xi’an Jiaotong University, Xi’an, 710061 China; 2 College of Forensic Medicine, Xi’an Jiaotong University, Xi’an, 710061 China; 3 Xi’an Chest Hospital, Xi’an, China; 4 Faculty of Veterinary and Animal Sciences, Muhammad Nawaz Shareef University of Agriculture, Multan, Pakistan; 5 The Second Affiliated Hospital of Xi’an Jiaotong University, Xi’an, China

**Keywords:** Microbiology, Molecular biology

## Abstract

Once the body dies, the indigenous microbes of the host begin to break down the body from the inside and play a key role thereafter. This study aimed to investigate the probable shift in the composition of the rectal microbiota at different time intervals up to 15 days after death and to explore bacterial taxa important for estimating the time since death. At the phylum level, Proteobacteria and Firmicutes showed major shifts when checked at 11 different intervals and emerged at most of the postmortem intervals. At the species level, *Enterococcus faecalis* and *Proteus mirabilis* showed a downward and upward trend, respectively, after day 5 postmortem. The phylum-, family-, genus-, and species-taxon richness decreased initially and then increased considerably. The turning point occurred on day 9, when the genus, rather than the phylum, family, or species, provided the most information for estimating the time since death. We constructed a prediction model using genus-level data from high-throughput sequencing, and seven bacterial taxa, namely, *Enterococcus*, *Proteus*, *Lactobacillus*, unidentified *Clostridiales*, *Vagococcus*, *unidentified Corynebacteriaceae,* and *unidentified Enterobacteriaceae*, were included in this model. The abovementioned bacteria showed potential for estimating the shortest time since death.

## Introduction

In forensic autopsies, the time since death refers to the time that has passed since the actual death. Postmortem interval (PMI) estimation plays a critical role in the investigation of abnormal death. Some methods have been described for PMI estimation, which include forensic entomology, purely physical methods, combined physical and chemical methods, cadaver self-degradation, and corruption-based methods^1^. The abovementioned methods can offer an estimation of PMI; however, each method is restricted in its fitness for use and accuracy depending on the environmental conditions and timescale of PMI (e.g., days, months) of a specific case^2–4^. Recently, the estimation of the postmortem interval using the postmortem microbiome has received much attention^2^. However, utilizing microorganisms to infer the PMI and time since death remains underexplored, and thus, it has become an area of considerable research in forensic science.

It is well accepted that the microbiota plays an important role in vertebrate post mortality decomposition^5–7^. A carcass undergoes several stages of decomposition, and enteric microbes facilitate decay by digesting macromolecules to produce gases, especially from the fresh stage to the rupture stage^1,2,8^. During the fresh stage, macromolecules are released from cells shortly after death, and the microbiota facilitates this process, breaking down macromolecules to simple molecules^6,7,9^. During the early stage, the microbiota performs anaerobic respiration, which yields a number of gases leading to cadaver bloats, and the microbial community experiences a major shift from anaerobic to aerobic conditions at the end of this stage^2,9,10^. During advanced decomposition, rupture of the abdominal cavity and exposure to the outside results in a shift in the bacterial community^2,9^. Therefore, vertebrate mortality may be associated with a predictable succession of microbes and can be used for estimating PMI. Clarifying the succession of the microbiota during decomposition has applications in forensic science, especially for identifying characteristic bacteria that are closely related to time since death at specific time points. Recent studies have reported bacterial community changes related to animal carcass decomposition^2,11–14^. Such studies are starting to determine the universal changes in bacterial communities, including changes in genera and families, identifying the most informative taxonomic microbial communities that change after death transmigration. These may be useful in providing PMI estimates and the relative abundance of bacterial communities that can be included in a useful model. Additionally, Metcalf et al.^2^ and Burcham et al.^13^ selected mice as a model; the former study aimed to understand microbial community changes during a 48-day decomposition process, while the latter investigated how changes in specific bacteria can potentially be used in time-since-death estimations. These studies collectively suggested that the reproducibility of microbial community succession during vertebrate postmortality decomposition can be used to predict PMI and warrants further study.

Machine learning is a robust tool for identifying trends in complex data, and it is usually used on microbiome data to identify succession in the microbiota^5^. In a study conducted by Pechal et al*.*, the researchers exposed pigs to an environment without human intervention and established a data model with 10 bacteria at the family level to explain the 94.4% of the time since placement of carcasses within 2–3 h. Another study demonstrated that microorganisms could be used to estimate the time since death within 3 days after death^2^. In 2020, researchers found that bacterial communities displayed significant differences between time of death and advanced decay and used machine learning to construct an integrated model for estimating PMI^14^.

Metcalf et al. constructed a model called the “microbial clock” that was capable of estimating a 48-day PMI with only 3 days of error^2^; therefore, taking advantage of statistical methods to construct usable and efficient models to estimate PMI might be a practicable alternative.

The principal purpose of studying the microbiome after death is to assist in death surveys by offering a consistent method to estimate PMI. In this study, 88 intestinal microbiota samples that were collected at 11-time points were sequenced using the IonS5^TM^XL platform to determine the characteristic microbiota, and then the main metabolic functions at each time point were assessed. This study aimed to determine the characteristic microbiota and use the bacterial community that contributed most to the decomposition time of rat carcasses to develop a statistical model for time-since-death estimation.

## Results

### Visible decay progression

The decomposition process of rat carcasses over 15 days was recorded and classified into five stages: the fresh stage began at 0.0 ± 0.0 h (mean ± SD) with no odor emitted; the bloat stage started with body expansion and the emission of, giving off odorous gases at 2.6 ± 1.1 days; the active stage started at 5.0 ± 1.0 days with the body being ruptured by accumulated gases and several parts of the tissues broken down, along with large amounts of liquid flowing out; the advanced stage began at 8.0 ± 1.0 days along with most parts of the tissues being removed; the dry stage occurred at 12.5 ± 1.9 days with no soft tissue remaining.

### Relative abundance of the gut microbiota in different groups

**Figure 1:**
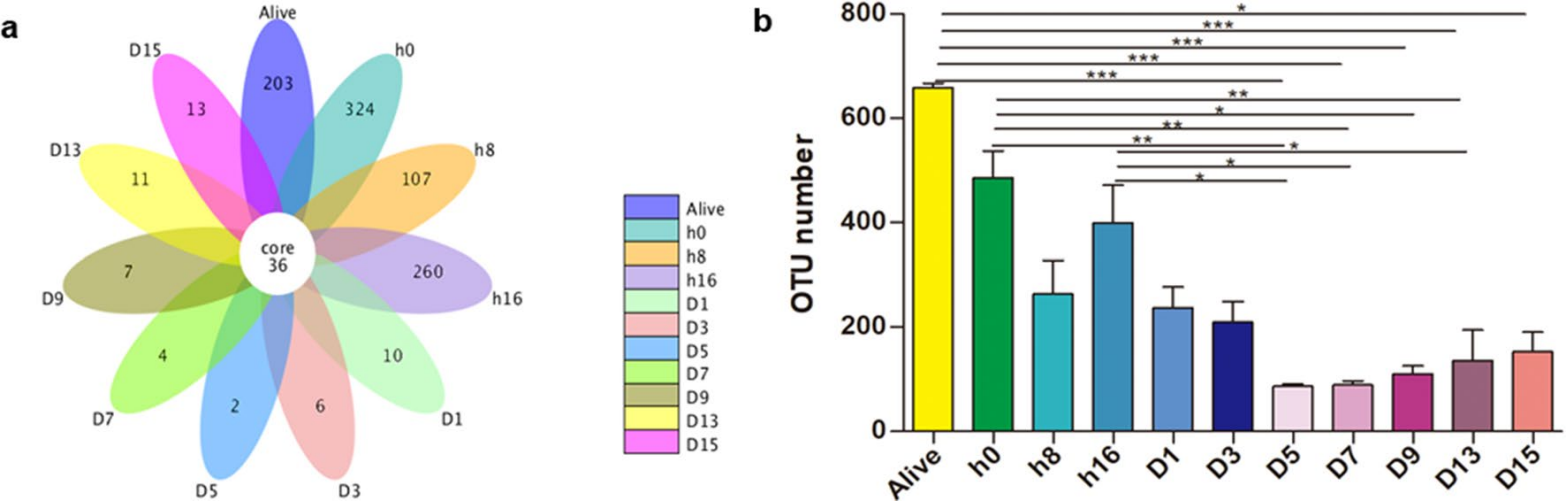
Shared operational taxonomic unit (OTU) analysis among 11 groups. Venn diagram of the microbial communities based on OTUs at 3% distance (**a**). A significant difference in OTU number among all time points was determined by the Kruskal–Wallis test with Bonferroni correction (**b**). *p < 0.05, **p < 0.01, ***p < 0.0001. Venn diagram was created by R (v 3.0.3)^15^.

A total of 7,029,815 raw and 6,674,323 clean reads were obtained by performing high-throughput sequencing, with an effective rate of 94.97% (the ratio of clean to raw reads). A total of 22,625 OTUs were identified based on 97% similarity, with an average of 257 OTUs per sample. The total usable sequences were classified into 33 phyla, 49 classes, 108 orders, 203 families, 465 genera, and 306 species. The species accumulation boxplot and rarefaction curves of all the samples were smooth as the number of sequences increased, demonstrating that this sequencing depth could mirror the complete bacterial species richness among the samples (see Supplementary Fig. S1 online). The basic information regarding the number of OTUs and alpha diversity indices in individual rats is shown in Supplementary Table S1 (see Supplementary Table S1 online). A Venn diagram was plotted to compare the similarities and variances among the communities obtained in the different groups. The eleven groups showed communities of 36 OTUs that were shared, with unique OTUs constituting84.94%, 90.00%, 74.83%, 87.84%, 21.74%, 14.29%, 5.26%, 10.00%, 16.28%, 23.4%, and 26.53% of the total at time points corresponding to alive, 0 h, 8 h, 16 h, 1 day, 3 days, 5 days, 7 days, 9 days, 13 days, and 15 days, respectively (Fig. 1a). The obtained OTUs for rectal microbes in the D5, D7 and D13 groups were significantly lower than those in alive (adjusted *p* values = 0.0001, 0.0001, < 0.0001, respectively), h0 (adjusted *p* values = 0.0040, 0.0036, 0.0030, respectively), and h16 (adjusted *p* values = 0.0461, 0.0422, 0.0353, respectively) groups, suggesting that bacterial richness decreased noticeably after rupture of rat carcasses (Fig. 1b). The core microbiome is represented by the overlapping areas of the circles in the Venn diagram, and 36 core members were identified in a similar environment. At different time points, 26 and 11 of the core members could be classified at the genus and species levels, respectively. *Enterococcus*, *Proteus*, *Lactobacillus*, *Bacteroides* were the most common genera, and the corresponding species were *Enterococcus faecalis*, *Proteus mirabilis*, *Lactobacillus reuteri* and *Bacteroides fragilis* (Fig. 1a).

### Microbial analysis at different levels

**Figure 2:**
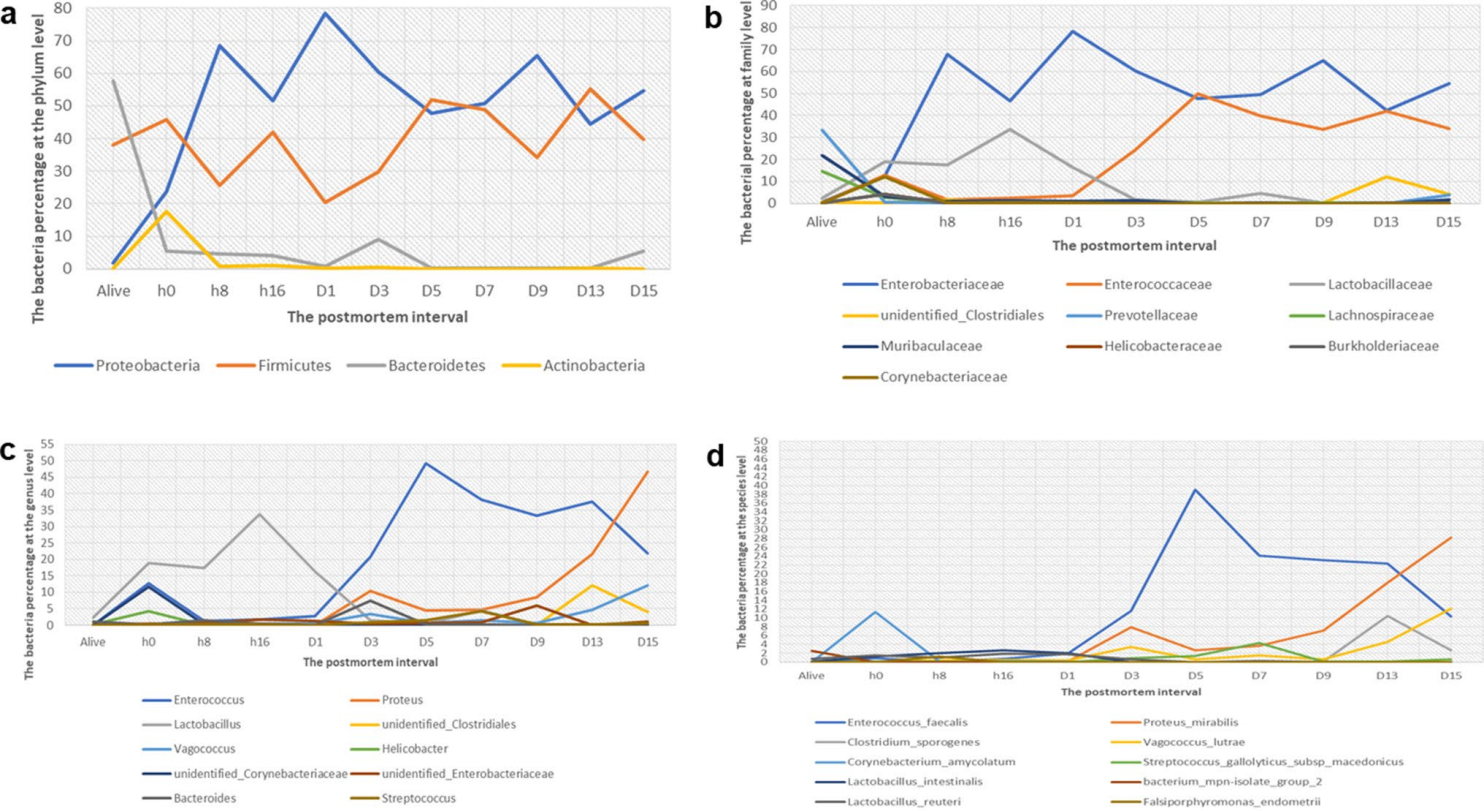
Line charts exhibit obvious shifts in microbial percentages at the phylum (**a**), family (**b**), genus (**c**), and species (**d**) levels.

**Figure 3:**
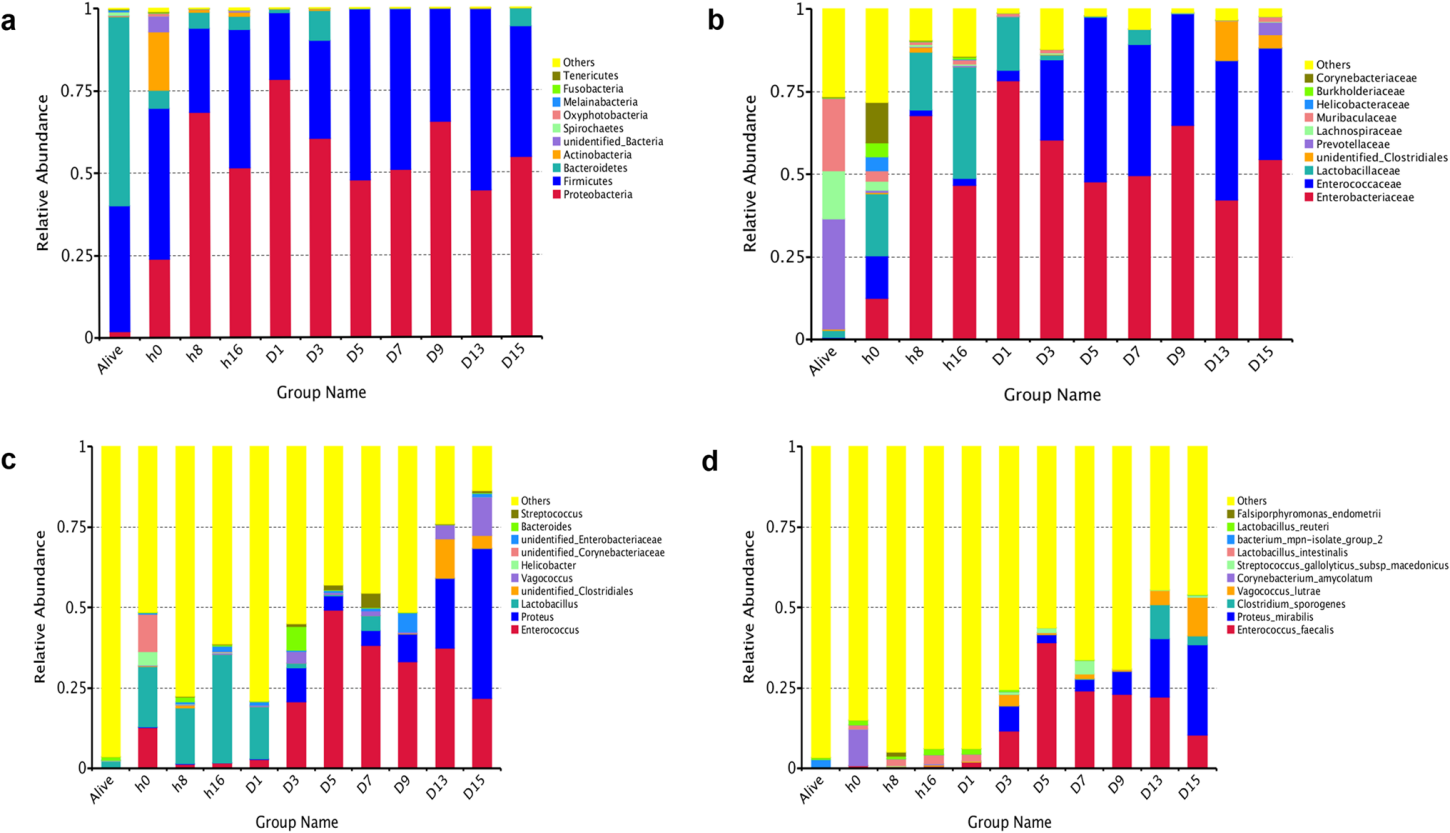
Taxonomic profiles of the rectal bacteria at eleven time points. Values represent the relative abundance of the top 10 phyla (**a**), the top 10 families (**b**), genera (**c**) and the top 10 species (**d**) present at eleven time points.

Data on the microbial community structure and predominant populations in the rectum were obtained by sequencing, and this information was analyzed at the phylum, family, genus and species taxonomic levels. The notable tendencies and fluctuations exhibited the relative richness of the diverse bacterial taxa in the rat cadaver rectum through the decomposition process, and the details are provided in the paragraphs below (Figs. 2; 3; Table 1).

**Table 1:** Significant differences in the abundance of OTUs at the phylum, genus and species levels in pre- and postmortem.

Bacteria	Mean relative abundance										
	Alive	h0	h8	h16	D1	D3	D5	D7	D9	D13	D15
**Phylum**											
*Firmicutes*	0.381422	0.458315	0.255179	0.420059	0.202931	0.298493	0.519011	0.487333	0.343126	0.551232	0.397023
*Proteobacteria*	0.018967^a^	0.238017^b^	0.685157^b^	0.515944^b^	0.783757	0.603994^b^	0.477884	0.509146^b^	0.655225	0.445179	0.546184^b^
*Bacteroidetes*	0.575744^a^	0.055408^b^	0.046913^b^	0.039948^b^	0.009058	0.090197^b^	0.002348	0.003179^b^	0.000968	0.001218	0.055198^b^
*Actinobacteria*	0.00136^a^	0.176496	0.008813	0.011762^b^	0.002587	0.00605	0.000186	0.000137^b^	0.000298	0.001174	0.000166^b^
**Genera**											
*Enterococcus*	0.000381^a^	0.127666^b^	0.013376^b^	0.016927^b^	0.028338	0.207753^b^	0.491857	0.382742^b^	0.332367	0.374643	0.217481^b^
*Proteus*	0.000049^a^	0.00023	0.000577	0.000455	0.000597^i^	0.104454^bj^	0.044976	0.046492^b^	0.084841	0.216454	0.466327^b^
*Lactobacillus*	0.022459^a^	0.189569	0.174197	0.336959^b^	0.162977	0.015514	0.003732	0.045177	0.001712	0.000176	0.000616^b^
*Vagococcus*	0.00001^a^	0.00047^b^	0.003551^b^	0.004299	0.003737	0.03471^b^	0.005624	0.014966^b^	0.00558	0.045661	0.120857^b^
*Helicobacter*	0.001624^a^	0.041934	0.000572	0.00201	0.000059^i^	0^bj^	0	0^ bp^	0	0.000005	0.000044^bq^
**Family**											
*Enterobacteriaceae*	0.005219^a^	0.123831^b^	0.67744^b^	0.466532^b^	0.782832	0.603554^b^	0.477766	0.495853^b^	0.648691	0.423356	0.543934^b^
*Enterococcaceae*	0.000391^a^	0.128634^b^	0.017162^b^	0.021926^b^	0.032519	0.243485^b^	0.497574	0.397865^b^	0.338118	0.420764	0.338612^b^
*Lactobacillaceae*	0.022488^a^	0.189569^b^	0.174847	0.336959^b^	0.162977	0.015514	0.003732	0.045177	0.001712	0.000176	0.000626^b^
*unidentified_Clostridiales*	0.003888^a^	0.003614	0.014624	0.000758^b^	0.000112^i^	0.000064^b^	0.000064	0.000245^b^	0.000528	0.121606	0.04012^j^
*Prevotellaceae*	0.334259^a^	0.007062^b^	0.002661^b^	0.003761^b^	0.000606	0.000377^b^	0.000083	0.000117^b^	0.00024	0.000064	0.037239^b^
*Lachnospiraceae*	0.145522^a^	0.025819^b^	0.005629^b^	0.005219^b^	0.001423	0.004651^b^	0.000161	0.000093^b^	0.000166	0.000068	0.002308^b^
*Ruminococcaceae*	0.186175^a^	0.050806^b^	0.010995^b^	0.014247	0.003262	0.006255^b^	0.000142	0.000103^b^	0.000445	0.000298	0.0076^b^
*Corynebacteriaceae*	0.000401^a^	0.122266^b^	0.001937	0.003218	0.00043^i^	0.000034^b^	0.00001	0^b^	0	0.00001	0^bj^
*Muribaculaceae*	0.217833^a^	0.03204^b^	0.009361^b^	0.012594^b^	0.007659	0.011718^b^	0.000034	0.000147^b^	0.000117	0.000083	0.015132^b^
**Species**											
*Enterococcus_faecalis*	0.000059^a^	0.008583^b^	0.001355	0.006862^b^	0.01947	0.115879^b^	0.39001	0.241289^b^	0.230305	0.22268	0.103055^b^
*Proteus_mirabilis*	0.000034^a^	0.000127	0.000372	0.000372	0.00045^i^	0.079285^b^	0.026675	0.036319^b^	0.071259	0.180374	0.281585^bj^
*Clostridium_sporogenes*	0^a^	0	0.000352^b^	0	0^i^	0.000024^b^	0.000044	0.00021^b^	0.000386	0.104938	0.027144^bj^
*Corynebacterium_amycolatum*	0.000044^a^	0.000665^b^	0.001585	0.001047	0.000181^i^	0.008432^b^	0.014164	0.043347^b^	0.001477	0.00114	0.006735^bj^
*Lactobacillus_intestinalis*	0.00045^a^	0.012618^b^	0.0206	0.027188^b^	0.019549	0.000298	0.000034	0.000088	0.000015	0^t^	0.000054^bu^
*Bacterium_mpn-isolate_group_2*	0.025628^a^	0.000044^b^	0.000034^b^	0.000015^b^	0.000015	0.000005^b^	0	0^b^	0	0	0.000005^b^
*Lactobacillus_reuteri*	0.006877^a^	0.014628	0.00941	0.018869	0.017822	0.006383	0.000191	0.002005	0.000127	0.000015	0.000196^b^

The significant findings were tested with MetaStat. *p* values were corrected for multiple testing (FDR-adjusted *q* < 0.05). “a, i, t” represent alive, h0, D1 and D13 pre- and postmortem compared with other time points, and “b, j, u” indicates a significant difference (*p* < 0.05 and *q* < 0.05).

At the phylum level, a total of 33 phyla were sequenced; among the top 10 phyla, Firmicutes, Proteobacteria, Bacteroidetes, and Actinobacteria were found to be present at all the time points. Bacteroidetes (54.57%), Firmicutes (45.83%), and Proteobacteria were the dominant phyla in the living samples at 0 h postmortem and at other time points. Proteobacteria showed three peak values within 15 days, at 8 h, D1 and D9. Interestingly, valleys appeared for Firmicutes at the same time, suggesting that bacteria belonging to these two phyla might play opposite roles in promoting carcass decomposition. Bacteroidetes decreased dramatically after death (from 57.57 to 5.54%), and then increased slightly at day 3 and day 15 postmortem. Changes in the conditions of rectal bacteria in the living samples had an impact on microbial community structure, particularly on the phylum Bacteroidetes. The relative abundance of Proteobacteria in the living samples was much lower than that at 0 h (*q* = 0.0064), 8 h (*q* = 0.0049), 16 h (*q* = 0.0042), 3 days (*q* = 0.0038), 7 days (*q* = 0.0031), and 15 days (*q* = 0.0151) postmortems, while the relative abundance of Bacteroidetes was much higher in the living samples than at the same points postmortem. The *q* values were 0.0023, 0.0049, 0.0042, 0.0037, 0.0031, 0.0042, respectively, and the relative abundance of Actinobacteria was significantly higher in the alive group than that at h 16 (*q* = 0.0408), day 7 (*q* = 0.0031), and day 15 (*q* = 0.0042) postmortems.

**Figure 4:**
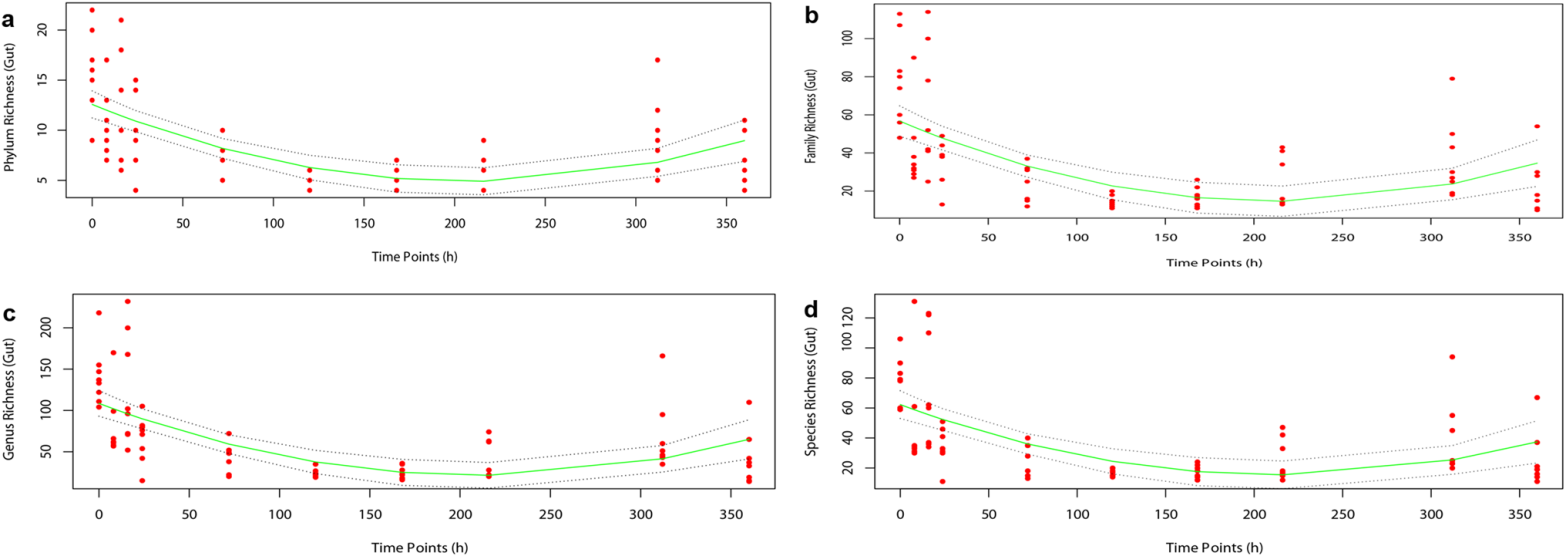
Gut bacterial community richness variation rules at the phylum, genus and species levels during the decomposition process. (**a**) At the phylum level, the bacterial community abundance decreased by 52.38% and then increased by 49.95%; (**b**) at the family level, the bacterial community abundance decreased by 56.74% and then increased by 31.17%; (**c**) at the genus level, the bacterial the bacterial community abundance decreased by 78.36% and then increased by 66.64%; (**d**) at the species level, taxon bacterial community decreased by 81.27% and then increased by 57.01%.

At the phylum level, there was an obvious change in bacterial richness following rat carcass decomposition, decreased by 52.38% from 0 h to day 9 (216 h) first and then increasing by 49.95% from day 9 to day 15 (360 h) (y = –0.0737x + 0.0002x^2^ + 12.5770, *R*^*2*^ = 0.390, *P* = 5.474e−09) (Fig. 4a).

At the family level, *Prevotellaceae*, *Muribaculaceae* and *Lachnospiraceae* were present at living samples as the most abundant families, while their abundance declined significantly at 0 h (*q* = 0.0025, 0.0025 and 0.0025), 8 h (*q* = 0.0088, 0.0089 and 0.0088), 16 h (*q* = 0.004, 0.004 and 0.004), days 3 (*q* = 0.0067, 0.0067 and 0.0067), days 7 (*q* = 0.0053, 0.0053 and 0.0053) and days 15 (*q* = 0.0078, 0.0116 and 0.0078) after death. As decomposition progressed, the abundance of *Lactobacillaceae*, *Enterobacteriaceae* and *Enterococcaceae* increased greatly, with these bacteria representing the major proportion from 0 h to 15 days.

At the family level, from 0 h to 15 days, there was a distinct change in bacterial richness following rat carcass decomposition, decreasing by 56.74% from 0 h to day 9 (216 h) first and then increasing by 31.17% from day 9 to day 15 (360 h) (y = –0.03950x + 0.0009x^2^ + 56.734, *R*^*2*^ = 0.356, *P* = 4.307e-08) (Fig. 4b).

At the genus level, *Lactobacillus* and *Enterococcus* appeared as the dominant genera at days 1 and 3–13 postmortem, respectively, *Helicobacter* was absent at days 7, 9, and 15 postmortem, and *Proteus* was the most abundant at day 15 postmortem. The relative abundances of *Enterococcus* and *Vagococcus* in the living samples were significantly lower than thoes at 0 h (*q* = 0.0026 and 0.0124), 8 h (*q* = 0.0073 and 0.0137), day 3 (*q* = 0.0079 and 0.0079), day 7 (*q* = 0.0064 and 0.0064), and day 15 (*q* = 0.0086 and 0.0086) postmortem; nevertheless, the *Helicobacter* abundance was much higher in the living samples than in the samples than in the samples at days 3 (*q* = 0.0079), 7 (*q* = 0.0063), and 15 (*q* = 0.0225) postmortem. The proportion of *Proteus* was significantly lower in the living samples than in the samples at days 3 (*q* = 0.0079), 7 (*q* = 0.0063), and 15 (*q* = 0.0086) postmortem, while *Lactobacillus* exhibited the opposite result at 16 h (*q* = 0.0106) and day 15 (*q* = 0.0086). This result suggested that beneficial bacteria in the rectum decreased postmortem, while opportunistic pathogens increased rapidly for adaptation to the change in living conditions, such as disruption of the oxygen supply caused by interruption of blood flow.

At the genus level, there was a significant shift in bacterial richness during the 0 h to day 15 decomposition period (Fig. 4c). The genus richness presented downward (78.36%) and upward (66.64%) trends, and the lowest time point was observed at day 9 (y = –0.8260x + 0.0020x^2^ + 108.6, *R*^*2*^ = 0.384, *P* = 7.881e-09).

At the species level, among the top ten species that existed at 8 h postmortem, *Clostridium sporogenes* and *Falsiporphyromonas_endometrii* exhibited the lowest relative abundances before 1- day postmortem and after 3 days postmortem, respectively. *E. faecalis* and *P. mirabilis* appeared during the whole 15-daydecomposition process after death; however, the former showed a downward trend with the proportion changing from 39.00 to 10.31% after 5 days, while for the latter, the proportion increased from 2.67 to 28.16%. The crossing point at approximately 20.00% occurred at a time point between days 13 and 15, after which *P. mirabilis* was more abundant than *E. faecalis*. The relative abundance of *E. faecalis* at 0 h (*q* = 0.0037), 16 h (*q* = 0.0043), and 3 (*q* = 0.0083), 7 (*q* = 0.0061), and 15 (*q* = 0.0065) days after death was much higher than that in alive samples, while the abundance of *Bacterium mpn isolate group 2* was substantially lower (*q* < *0.05*). The relative abundance of *C. amycolatum* was markedly higher in alive samples than in samples at 0 h (*q* = 0.0037) and 3 (*q* = 0.0083), 7 (*q* = 0.0061), and 15 (*q* = 0.0065) days postmortem, and the abundance of *Lactobacillus reuteri* and *L. intestinalis* were significantly higher in the alive group than that at 15- days (*q* = 0.0065 and 0.0301).

The species taxon richness first decreased (81.27%) and then increased (57.01%) as decomposition progressed and the turning point appeared at day 9 (y = –0.4382x + 0.0010x^2^ + 62.2575, *R*^*2*^ = 0.339, *P* = 1.214e-07) (Fig. 4d).

### Characterization of bacterial diversity and community structure

The complete rectal microbiota community was evaluated based on diversity and richness, which were calculated at 97% similarity. The alpha diversity index of the Shannon index for the rectal bacteria in the living samples was considerably higher than that in the samples at 5 (adjusted *p* = 0.0111), 7 (adjusted *p* = 0.0540), 9 (adjusted *p* = 0.0407), 13 (adjusted *p* = 0.0095), and 15 (adjusted *p* = 0.0092) days postmortem, suggesting that the diversity of the bacterial community in the rectum decreased noticeably after day 5 postmortem compared with that of the alive group (Table 2). The richness indices (ACE and Chao 1) decreased slightly before day 5 but decreased substantially after day 5. These results suggested that day 5 may be an important turning point for microbial community composition structure. All the alpha diversity indices are presented in Table 2.

**Table 2:** Alpha diversity index obtained by high-throughput analysis of intestinal microbial richness and diversity.

Time points	Observed_species	Shannon	Simpson	Chao1	ACE	Goods_coverage	PD_whole_tree
Alive	574^a^	6.284^a^	0.957^a^	681.781^a^	691.263^a^	0.995^a^	47.217^a^
h0	410^c^	4.845	0.842	472.923^c^	474.917^c^	0.997	45.098
h8	191	2.77^b^	0.681^b^	256.809	276.089	0.998	29.156
h16	321^e^	3.67	0.772	393.452^e^	414.277^e^	0.997^e^	54.33^e^
D1	159	2.414^b^	0.665^b^	238.27	269.216	0.997	23.572
D3	167	3.316	0.781	210.444	214.123	0.998	20.155
D5	61^bdf^	2.437^b^	0.658^b^	80.908^bdf^	92.725^bdf^	0.999^bf^	14.241^bf^
D7	64^bd^	2.633	0.739	78.199^bdf^	90.613^bdf^	0.999^bf^	10.862^bf^
D9	81^bd^	2.629^b^	0.705	107.505^bd^	114.157^bd^	0.999^bf^	13.614^bf^
D13	102^b^	2.388^b^	0.656^b^	148.395^b^	163.889^b^	0.998^b^	17.613^b^
D15	103^bd^	2.60^b^	0.697^b^	134.112^bd^	139.958^bd^	0.999^bf^	11.867^bf^
P*	< 0.0001	< 0.0001	0.0011	< 0.0001	< 0.0001	< 0.0001	< 0.0001

The values showed in the table are the mean values of each group; significant differences were determined with the Kruskal–Wallis test for Dunn's multiple comparison test by GraphPad Prism*. p* values were corrected by Bonferroni correction. “a, c, e” represents alive, h0, and h16 pre-and postmortem compared with other time points, and “b, d, f” indicates a significant difference.

**Figure 5:**
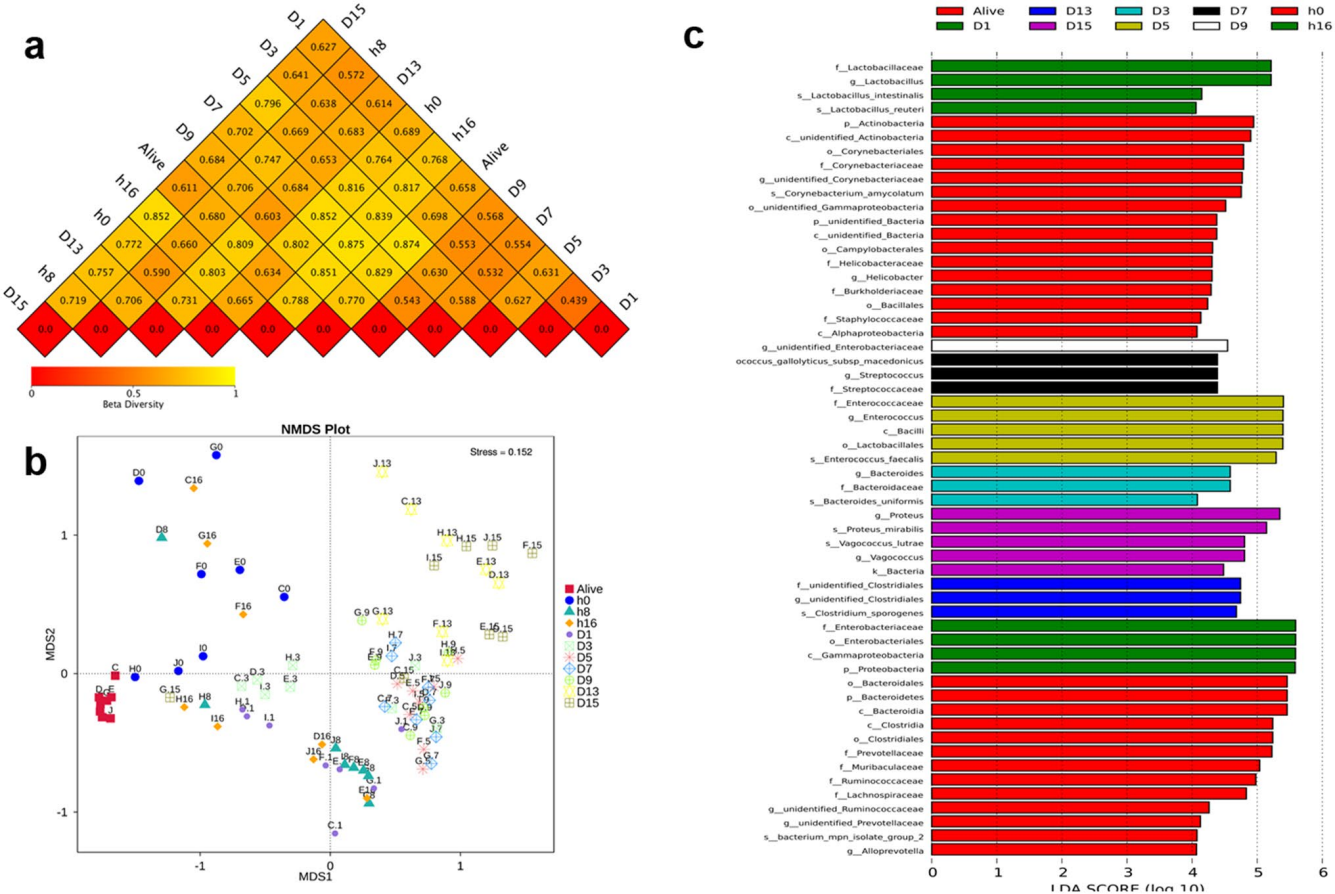
Beta diversity of bacterial populations at eleven-time points reflects intergroup differences. A heatmap was drawn by using the unweighted UniFrac distance (**a**). The microbial diversity at a certain time point increased, followed by the size of the value. NMDS (nonmetric multidimensional scaling) coordination plot among the whole samples (**b**). A stress value less than 2 indicates that NMDS can accurately reflect the difference between samples. Significant taxa obtained at the sampling time points using LEfSe analysis. Linear discriminant analysis (LDA) plots of bacteria at different levels for all time points (**c**). NMDS was performed by R (v 2.15.3)^16^.

The similarities in the gut microbiota communities of rats among the eleven groups were estimated using beta diversity metrics, such as NMDS and beta diversity heatmaps. As presented in Fig. 5a, the differences in coefficients among all the groups were higher than 0.5, indicating that the bacterial community in different groups exhibited great diversity. All the samples were clustered into 11 prime clusters. According to the NMDS (stress = 0.152), the bacterial communities of the rectum samples were separated into three clusters between the late and early PMI (Fig. 5b). Notably, the 8-h postmortem interval could be significantly separated from the other groups, indicating that the microbiota at 8 h after death differed from the other two clusters.

LEfSe is a biomarker detection and description tool for performing high-dimensional statistical analyses. LEfSe analysis was performed to compare the projected bacterial community among the 11- time intervals at different levels (Fig. 5c). The results of this analysis suggested that the abundance of related taxa was considerably diverse among all the groups. The LDA scores revealed that the relative abundances of *C. amycolatum*, *Entero isolate group 2, Bacteroides uniformis*, *E. faecalis*, *Streptococcus gallolyticus subsp macedonics*, and *C. sporogenes* were substantially higher at postmortem intervals of 0 h, 1 day, 3 days, 5 days, 7 days, and 13 days, respectively, than in the remaining groups, while *P. mirabilis* and *V. lutrae* were more abundant on day 15 postmortem.

### Constructing a model for PMI

The best subset selection was combined with phylum, family, genus, and species indicators to construct the model that best explained the aberrance in time since death from 0 h to day 15 (see Supplementary Fig. S2, S3 online; see Supplementary Table S2 online). Supplementary Table S2 shows that the poorest model for PMI appeared at the phylum level (16.1% of the variation). Models constructed with family features were poor due to their higher *GCV* values. The taxon that contributed most to postmortem was identified at the genus level. The best subset selection results showed that seven bacteria at the genus level were selected as the best features to develop the model. These seven genera were: *Enterococcus*, *Proteus*, *Lactobacillus*, unidentified *Clostridiales*, *Vagococcus*, unidentified *Corynebacteriaceae*, and unidentified *Enterobacteriaceae*, and this model contained the most information and explained 87.2% (generalized cross-validation score (*GCV* = 0.307) of the variation in time of death in this study. The species, including four bacteria, were identified from best subset selection as the most informative and explained 56.6% (*GCV* = 0.515). This model was poorer in explaining the variation in time since death than the model constructed by the genus features.

### PICRUSt

**Figure 6:**
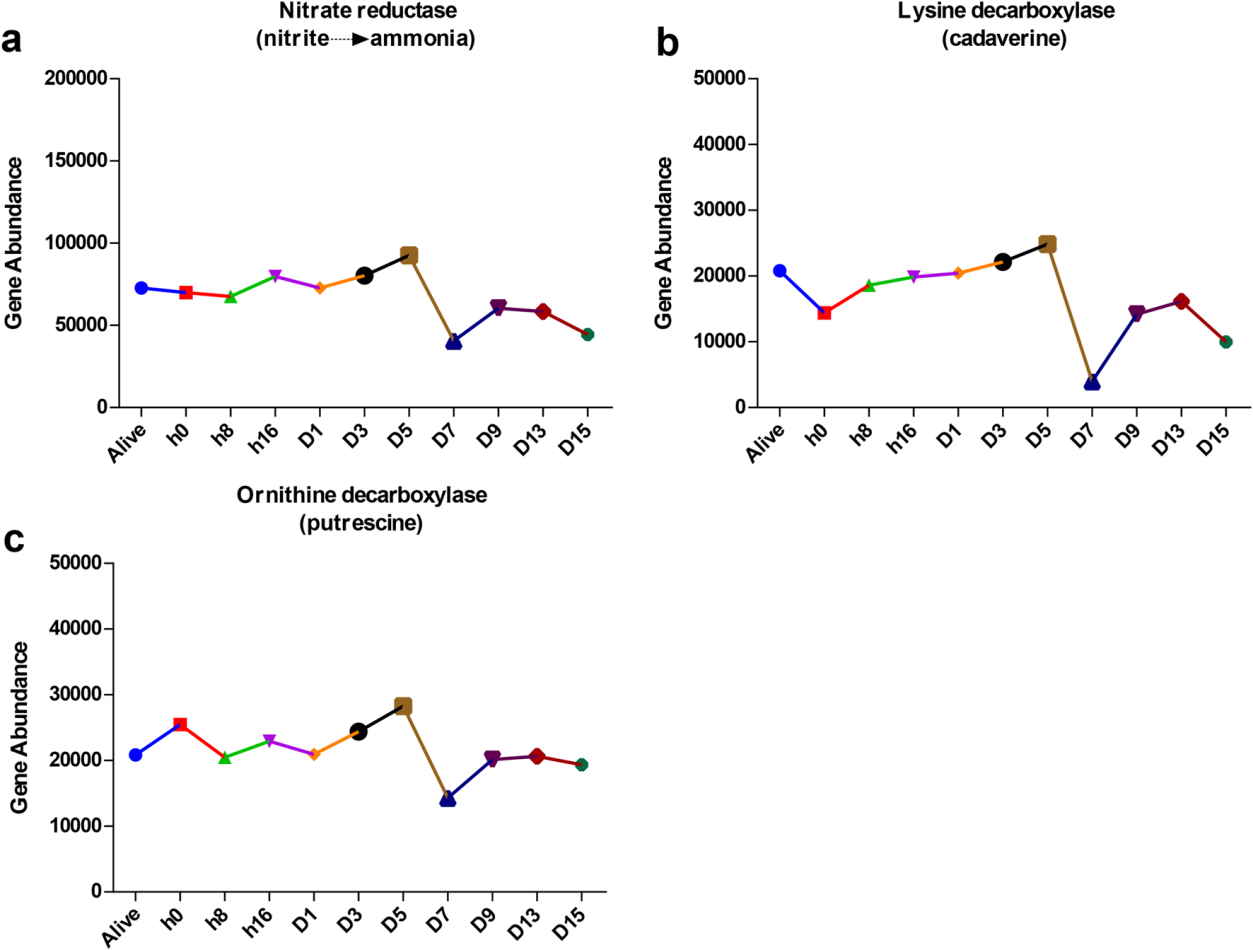
PICRUSt2-predicted enzyme-level genes in the gut of rats during decomposition process. (**a**) Nitrite reductase, (**b**) lysine decarboxylase and (**c**) ornithine decarboxylase.

The probable functions of the rectal microbiota of rats before and after death were inspected by PICRUSt2 based on the normalized OTU tables. A total of 418 pathways were identified across samples at 11- time points. Three genes associated with the breakdown of lysine and ornithine and reduction of nitrate, leading to the rotting smell associated with decomposition, attributed to the accessory substances cadaverine, putrescine and ammonia. We determined that these three genes involved in nitrogen cycling and amino acid degradation increased before day 5, but decreased markedly after day 5 (Fig. 6).

## Discussion

The microbiome composition in the living body is complex, and multiple significant differences have been observed between and within individuals^17^. Microorganisms exist both inside and outside cadavers and exhibit a distinct and temporal shift during the process of decay^18^. The gut, as an organ that contains a large number of bacteria, has been shown to exhibit variations during the decomposition of cadavers^9,19,20^, which prompted this study. If researchers want to use bacteria to infer the approximate postmortem interval, a database of the changes (in structure and composition) under different conditions during the cadaver degradation process should first be developed. This study aimed to construct a usable model to estimate the time since death and examine the bacterial composition and structure under indoor temperature (20.63 ± 0.93 °C) and humidity (15.37% ± 2.79%) conditions during a 15-day process of decomposition, thereby contributing to basic data accumulation for future use. Mammalian decomposition might involve a predictable succession of bacteria. The decomposition process could be repeated across individual hosts and conditions. Cadavers, as a concentrated source of nutrients in ecosystems for hundreds of millions of years, provide nutrition for the microbiota^21^. Moreover, a rat model provides convenience for evaluating microbial differences during decomposition and for sample collection^22^. Therefore, rats were selected as a model of human cadaver decomposition.

The bacterial communities in the decomposing rats showed a distinct shift in the structural composition and relative abundance compared with those in the living rats. Indices for evaluating bacterial community richness and diversity, such as observed species, Shannon, Chao1, and ACE indices, were significantly reduced after day 5 postmortem compared to those in the premortem samples, which was consistent with a previous study^20^. We also found that the richness indices (ACE and Chao 1) showed consistent variation before 9 days postmortem but displayed the opposite trend after 9 days, suggesting that decomposition of the rats led to an evident change in the internal environment on day 9 and that external environmental factors influenced the gut bacterial community structure and composition after day 9. During the rat decomposition process, on day 3, gas accumulation led to bloating and rupture of the animal carcass, replacing the internal conditions with the external conditions, which resulted in a decrease the anaerobes such as *Lactobacillus*, while the facultative anaerobe *Enterococcus* seized the opportunity to thrive.

This study indicated a notable variation in the gut bacterial diversity and relative abundance during the decomposition period of 15 days at the phylum, genus, and species levels. The results of this study showed that the main phyla in the intestinal samples that were present at high abundance before death were Firmicutes and Bacteroidetes, which was similar to the results of the NIH Human Microbiome Project^23^ (Fig. 3a). Although the dominant bacterial community diversity at the phylum level showed no evident fluctuation, we found that changes in the phyla Firmicutes and Proteobacteria were in opposite direction in terms of relative abundance over time, which was consistent with a previous study^24^. Previous research focused on the epinecrotic communities on human cadavers showed that Firmicutes was the inherent phylum in cadavers, while Proteobacteria was initially identified in the environment, and the reason for the increase in Proteobacteria was the migration of external bacteria^24–26^. This suggested that the increased relative abundance of Proteobacteria in the rectum of dead rats after 8 h may be due to migration from the external environment. At the genus level, we found that *Lactobacillus* was the dominant bacterium at the time points before day 3, while after day 3, the abundance of *Enterococcus* and *Proteus* increased, and these become the most abundant bacteria. This transition occurred at the beginning of the bloating stage with gas accumulation, through the rupture stage with fluids releases, and eventually to the dry stage in our study. A primary literature search showed that *Lactobacillus* has been shown to increase at the bloating stage but decreased greatly after the rupture stage, and *Proteus*, which is mainly related to sewage and animal matter becomes abundant^22,27^. Our results also provided support for these findings. The relative abundance of Bacteroidetes decreased notably after death, which was similar to the results of a previous analysis^6^. As previously reported^28–32^, the *Vagococcus* genus was likely to be isolated from marine hosts, contaminated food, severe skin infection wounds, and soil under a decaying pig carcass, and the high level might be attributed to the larvae of blowflies associated with cadavers, suggesting that *Vagococcus sp.* might facilitate further cadaver decomposition in our study. LEfSe results suggested that the seven bacteria at the species level were identified as seven potential PMI indicators. *B. uniformis* belongs to *Bacteroides spp*. and can be regarded as a PMI indicator at day 3 postmortem. It has been reported that^33^ *Bacteroides* spp*.* could be used as a quantitative indicator of PMI. *E. faecalis*, *Streptococcus gallolyticus subsp. macedonicus*, and *C. sporogenes*, from *Enterococcus*, *Streptococcus,* and *Clostridium* spp*.*, respectively, were also included and could also be used as PMI indicators on days 5, 7, and 13 postmortems, respectively; these species have previously been reported to be the most abundant species during decomposition^34^. Our study also showed an evident shift in *C. amycolatum* at the early postmortem stage, whereas *E. faecalis*, *P. mirabilis*, *C. sporogenes,* and *V. lutrae* were identified to be the most abundant in late postmortem intervals. Interestingly, the present findings on *P. mirabilis* and *V. lutrae* indicated almost parallel changes, although the percentages were different. The abovementioned two species of bacteria after day 15 postmortem showed a downward trend, while *E. faecalis* and *C. sporogenes* showed an upward trend. According to a previous study, *P. mirabilis* was able to attract blowflies, which was the reason for the high percentage of *V. lutrae*^35^.

We constructed a model that could explain 87.2% of the variation in the time of death within 1 h. For the corpse the decomposition process, the continuous variables for analysis and particularly fine model developed to estimate the postmortem interval were similar to those in a previous study^14^. The features containing most information for evaluating time of death were selected by best subset selection the genus level, and the features of the poorest model belonged to the phyla. Previous research identified that the poorest model for estimating physiological time for the epinecrotic bacterial community was composed of phylum features, which was consistent with our study^12^; however, the features of the best model were derived from the family. They did not incorporate the bacterial genera into the research, and the sampling position was different from ours. A study by Hunter et al. (2016) reported their focus on the human skin beneficial bacterial community to develop a promising tool for time of death estimation. Their results were similar to those of this study and indicate that the genus was the most informative taxon^36^. Therefore, variation in the community at the genus taxonomic level was the most promising indicator of the PMI and warrants further research. Additionally, a previous study constructed a model with ISA indicator taxa and generalized additional model at the family level that could explain 94.4% of the variation in physiological time, but in this model, overfitting may occur for the low abundance samples^12^. However, all of these models should be tested in different environments, with blind testing to verify whether such a model can be used^37^. Therefore, the next step for our study is to validate this model under field conditions with a larger quantity of samples at one time point.

Predictive functional analysis can provide potential insights into microbial functions. In this study, we found three categories of genes encoding enzymes, including nitrite reductase, lysine decarboxylase and ornithine decarboxylase. The foul-smelling byproducts ammonia, cadaverine and putrescine were generated by these enzymes from nitrite, lysine and ornithine. Microbiota as a main force in this process, such as nitrite reductase, lysine decarboxylase and ornithine decarboxylase were commonly found in *Enterobacteriaceae*^38,39^ and the ornithine decarboxylase test was positive in *P. mirabilis*. *Enterobacteriaceae*, as specific spoilage organisms, have great power to generate polyamines, especially cadaverine and putrescine^40–42^. Additionally, *P. mirabilis*, as a putrefying bacteria, was capable of breaking down urea to NH_3_ during decomposition, suggesting that *Enterobacteriaceae*, including *P. mirabilis*, played a key role in facilitating cadaver decomposition. Metabolic characteristics are altered due to decreased richness in the gut microbiome^43^. In this study, the richness decreased considerably before 5 days postmortem (Chao 1 and ACE), which was probably a main reason for the changes in functional genes.

Although the results of this study provide a detailed description of the bacterial composition within a decomposing cadaver system and suggest that microbial community data can be developed into as official indicators for estimating the PMI, additional research is required to better comprehend this process, such as the shifts in and role of fungi during carcass decomposition.

Taken together, the bacterial community exhibited a distinct shift in both composition and structure during the 15 days decay. Proteobacteria and Firmicutes exhibited opposite patterns throughout in the decomposition process in this study. Additionally, the bacterial genera were the most promising features for estimating PMI*.* Therefore, these findings offer a foundation for analysis of the bacterial community at specific time points after death.

## Methods

### Laboratory animals and their basic information

This study aimed to investigate the probable shift in the composition of the rectal microbiota at different time intervals up to 15 days after death and to explore bacterial taxa important for estimating the time since death. A total of eight healthy male Sprague Dawley rats were purchased from the Experimental Animal Center of Xi’an Jiaotong University with an average body weight of approximately 200–220 g/rat. The rats were sacrificed using the cervical dislocation method. The dead rats were placed in eight cages (0.46 × 0.30 × 0.16 m) and allowed to interact with the outside environment, except with insects. The experimental samples were collected from the same batch of living (one-time point) and dead (10 time points) rats. All animal handling protocols and treatments were approved by the Institutional Animal Use and Care Committee of Xi’an Jiaotong University, Shaanxi, China (Ethics Approval Number: 2017-388), and the methods were carried out in accordance with the approved guidelines.

### Method of sample collection and selection of experimental time points

Rectal samples were collected after the death of living rats. The postmortem putrefaction of all the dead rats took place in Xi’an city, Shaanxi Province, China (34°15′39.9″°N, 108°56′33.32″°E) in December 2017. All animal experiments were approved by the Institutional Animal Use and Care Committee of Xi’an Jiaotong University (No: 2017-388). Samples of the rectal microbiota from the dead rats were swabbed with sterile cotton swabs dipped in sterile saline for one minute before and after execution to obtain more rectal bacterial samples according to previously described protocols^6,9,12^. The time points of sample collection were as follows: before death; 0 h, 8 h, and 16 h after death; and 1 day, 3 days, 5 days, 7 days, 9 days, 13 days, and 15 days after death (the time points were designated alive, h0, h8, h16, D1, D3, D5, D7, D9, D13, and D15, respectively). Swabs were then placed in 1.5-mL sterile Eppendorf (EP) tubes and stored at − 80 °C until further use. The sampling period was from December 6–21, 2017. The average temperature was 20.63 °C ± 0.93 °C, and the humidity was 15.37% ± 2.79%, during sampling.

### DNA extraction

The total genomic DNA of the bacterial samples was extracted from the swabs using the QIAamp DNA Mini Kit (Qiagen, Germany), and the specific procedures were performed according to the manufacturer’s instructions. The DNA concentration was determined using a NanoDrop2000 (Thermo Scientific, Waltham, MA, USA), and the extracted DNA was stored in a refrigerator at − 80 °C until further use.

### High-throughput sequencing

#### Operation flow

Single-end sequencing (SE600) was performed on Thermo Fisher's IonS5^TM^XL sequencing platform to obtain high-throughput sequences. The total DNA extracted from the bacteria was amplified with the 16S rRNA V3 + V4 universal primers 341F (5′-CCTAYGGRBGCASCAG-3′) and 806R (5′-GGACTACNNGGTATCTAAT-3′). The amplified products were recovered, purified, and quantified, and then, the corresponding mixing ratio of each sample was adjusted according to the quantitative results. Thereafter, the library was prepared, and sequencing was performed using the sequencing platform.

#### Processing of the sequencing data

Cutadapt (v 1.9.1) software^45^ was used to remove ambiguous bases (N); organize the sequencing data according to the barcodes, remove low-quality bases (referring to sequences shorter than 400 bp or longer than 450 bp and those that included abundant “N” in the paired-end), barcodes, and primers, and then to obtain the raw data. UCHIME^46^ was used to remove chimeras of the sequences in the raw data, and then, high-quality sequences were obtained.

#### OTU clustering and species annotation

Uparse (v 7.0.1001) software^47^ was used to discard repetitive sequence from the high-quality sequences first, and then, the representative sequences were obtained. Second, we sorted the representative sequences according to the size of each sequence (the number of repeats) and removed sequences with size = 1. Finally, the OTU representative sequences were obtained by clustering according to 97% similarity of the sequences. The OTUs with the highest frequencies were selected as representative sequences. The Mothur method was used to analyze the species annotations using the SSUrRNA^48^ database of SILVA132^49^. MUSCLE (v 3.8.31) software^50^ was used for rapid multisequence alignment to obtain the phylogenetic relationships of all OTU sequences. The filtered OTUs were rarefied according to the number of sequences from the library with the lowest sequencing depth within each comparison group.

### Sample complexity analysis

Alpha diversity (Chao1, ACE, Shannon, and Simpson indices) was calculated using QIIME (v 1.9.1) software^51^, and the species accumulation curves were drawn using the Rarefaction Curve tool of R software (v 2.15.3). Alpha diversities were calculated based on the sample with the fewest sequences as a standard. Alpha diversity among the different groups was compared, and the differences were determined by performing the nonparametric Kruskal–Wallis test of Dunn's multiple comparison test. The results were considered statistically significant when the adjusted *P* was < 0.05 according to Bonferroni correction.

### Diversity comparison analysis

The UniFrac distance was calculated using QIIME (v 1.9.1) software, and hierarchical clustering of the samples was performed using the unweighted pair group method with arithmetic mean. The nonmetric dimensional scaling (NMDS) diagram was drawn using the vegan package of R software. The beta diversity index was analyzed using R software, and the nonparametric tests were subsequently performed subsequently. Linear discriminant analysis effect size (LEfSe)^52^ was used to identify the microbial taxa that were abundant in the gut at different successive of time points, which was based on an LDA score > 2.0 and *p* < 0.05. Analysis of similarities was performed by using the Adonis function of the vegan package of R software, and species analysis with significant differences between different groups was performed using R software.

### PICRUSt

Phylogenetic investigation of communities by reconstruction of unobserved states 2 (PICRUSt2) was used to predict metagenomic functions based on the normalized OTU tables^53^. PICRUSt2 predictions based on a comprehensive reference database of MetaCyc and the Enzyme Commission (EC) number databases for generating organism-specific pathways.

### Statistical analysis

All statistical analyses were performed using GraphPad Prism (v 6.02) software or the R (v 3.0.3) (v 3.6.3) and (v 2.15.3) packages^15,16,54^, and the Kruskal–Wallis test of the Dunn's multiple comparison tests was performed. An adjusted *p* value < 0.05 was considered statistically significant. MetaStat was used to test significant differences in microbiota abundance at different levels among groups to obtain *p* values, and then the *q* value was obtained by adjusting the *p* value (FDR Benjamini–Hochberg correction, *q* < 0.05). The leaps (v 3.1) package in R (v 3.6.3) (https://www.r-project.org/) was used to construct the best subset selection, and the mgcv (v1.8-31) package in R (v 3.6.3) was used to construct generalized additive model (GAMs)^54^.

## Supplementary Information

Supplementary Information

## Data Availability

The codes used in the current study are available from the corresponding author upon reasonable request (E-mail: xujiru@mail.xjtu.edu.cn) and the dataset generated during this study will be uploaded to http://www.ncbi.nlm.nih.gov/Traces/home/ after the paper is accepted.
