# Supplementary material for: Potential use of molecular and structural characterization of the gut bacterial community for postmortem interval estimation in Sprague Dawley rats
Source: Sci Rep. 2021 Jan 8;11:225. doi: 10.1038/s41598-020-80633-2 (PMC7794466; doi:10.1038/s41598-020-80633-2)
Supplement: Supplementary file 1 — Supplementary Information [file 41598_2020_80633_MOESM1_ESM.pdf]

# Potential Use of Molecular Characterization and Structure of the Gut Bacterial Community for Postmortem Interval Estimation in Sprague Dawley Rats

Huan Li [a], Siruo Zhang [a], Ruina Liu [b], Lu Yuan [a], Di Wu [b], E Yang [a], Han Yang [c], Shakir Ullah [a], Hafiz Muhammad Ishaq [d], Hailong Liu [e], Zhenyuan Wang [b**], Jiru Xu [a*]

a Department of Microbiology and Immunology, School of Basic Medical Sciences, Xi'an Jiaotong University, Xi'an, China

b College of Forensic Medicine, Xi'an Jiaotong University, Xi'an, China, 710061

c Xi'an Chest Hospital

d Faculty of Veterinary and Animal Sciences, Muhammad Nawaz Shareef University of Agriculture, Multan, Pakistan

e The Second Affiliated Hospital of Xi'an Jiaotong University, Xi'an, China

* Corresponding author:

Department of Microbiology and Immunology, School of Basic Medical Sciences, Xi'an Jiaotong University, Xi'an, China, 710061

Phone: +86-029-82657814

Fax: +86-029-82657814

E-mail: xujiru@mail.xjtu.edu.cn.

** Corresponding author:

College of Forensic Medicine, Xi'an Jiaotong University, Xi'an, China, 710061

Phone: +86- 029-82655472

Fax: +86- 029-82655472

E-mail: wzy218@xjtu.edu.cn

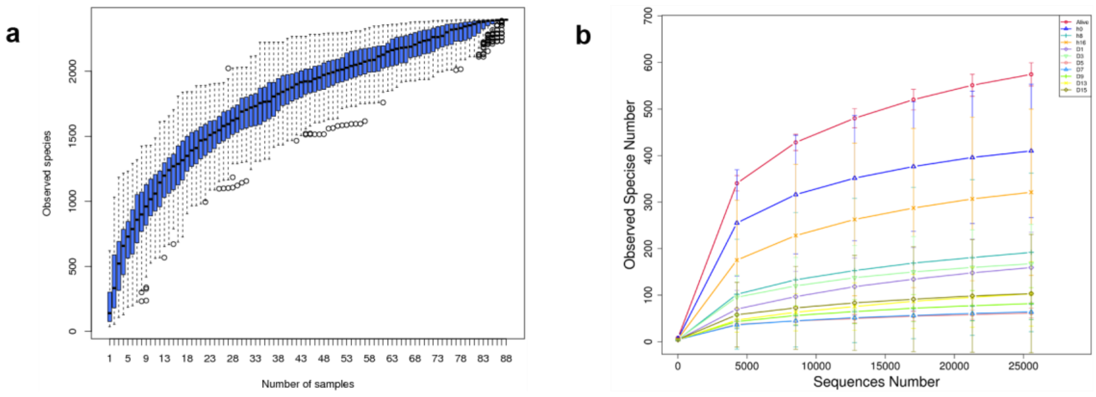


**Supplementary Figure S1** Species accumulation boxplot, evaluating whether sample size was sufficient or not and determining species richness (a). Rarefaction analysis of the microbiota of the rat rectum (b). Created with R (v 2.15.3)[1]

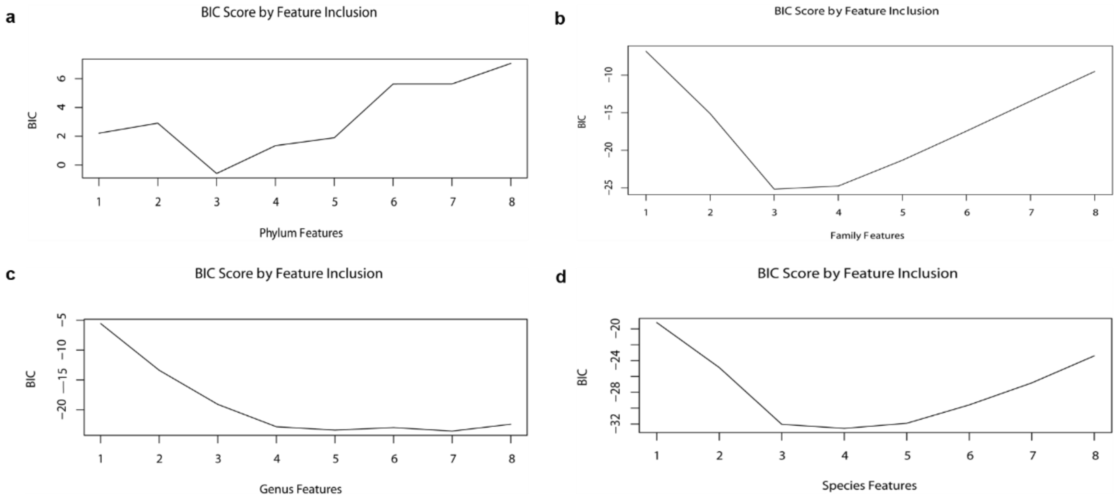


**Supplementary Figure S2** BIC (Bayesian Information Criterion) used for model and feature selection and the lower BIC value the better model and feature. (a) X-coordinate is number of phylum taxon features having important influence for PMI. Y-coordinate represents specific BIC value and 3 phylum taxon features are the ideal candidate for model development. (b) X-coordinate is number of family taxon features having important influence for PMI. Y-coordinate represents specific BIC value and 3 family taxon features are the ideal candidate for model development. (c) X-coordinate is number of genus taxon features having important influence for PMI. Y-coordinate represents specific BIC value and 7 genus taxon features are the ideal candidate for model development. (d) X-coordinate is number of species taxon features having important influence for PMI. Y-coordinate represents specific BIC value and 4 species taxon features are the ideal candidate for model development. Created with R (v 3.6.3)[2].

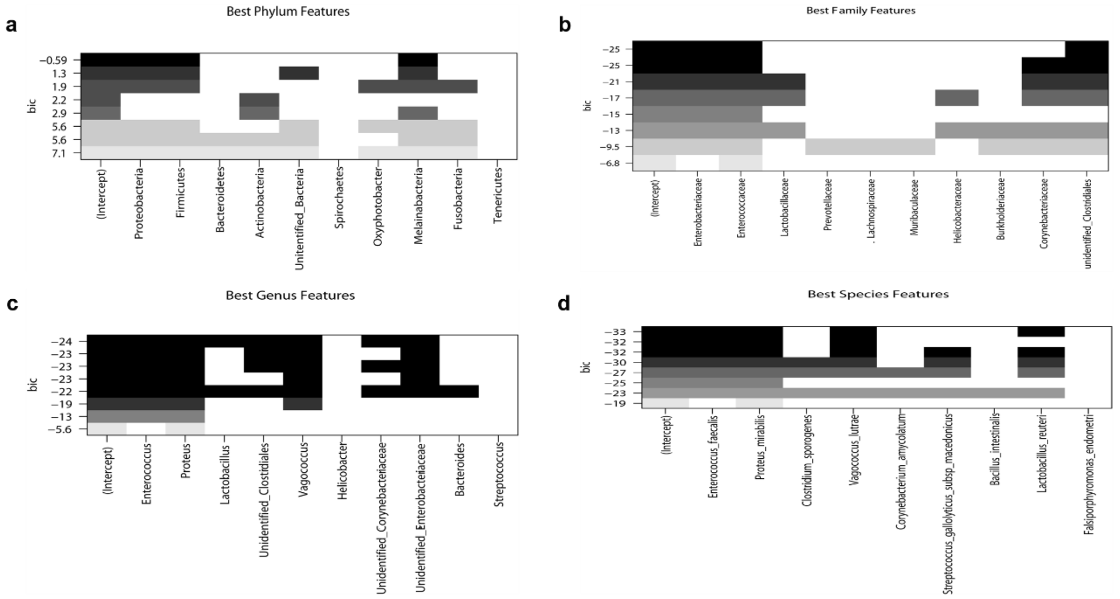


**Supplementary Figure S3** According to cumulative value of BIC (the darker the color, the smaller the BIC, the blank box means that BIC is zero), 3 best phylum features were selected which were Spirochaetes, Tenericutes, Oxyphotobacteria, and the corresponding BIC were 0, 0, 14.6 (a). According to cumulative value of BIC (the darker the color, the smaller the BIC), 3 best family taxon features were selected which were *Enterococcaceae*, *Enterobacteriaceae*, *unidentified_Clostridiales*, and the corresponding BIC were –110.5, –125.5, –132.3 (b). According to cumulative value of BIC (the darker the color, the smaller the BIC), 7 best genus taxon features were selected as the most contribution bacteria for PMI evaluation which were *Proteus*, *Enterococcus*, *Vagococcus*, *unidentified Enterobacteriaceae*, *unidentified Clostridiales*, *unidentified Corynebacteriaceae*, *Lactobacillus* and the corresponding BIC were –152.9, –147.3, -134, -115, -92, -69, - 46 (c). According to cumulative value of BIC (the darker the color, the smaller the BIC), 4 best species taxon features were selected which were *Proteus mirabilis*, *Enterococcus faecalis*, *Vagococcus lutrae*, *Lactobacillus reuteri*, and the corresponding BIC were –211, –192, –167, –135 (d). Created with R (v 3.6.3)[2]

**Supplementary Table S1** OTU number and Alpha diversity indexes in individual rat rectum

| Sample name | Time Points | Raw sequences | Clean sequences | OTUs | Shannon index | Observed species |
|---|---|---|---|---|---|---|
| C | alive | 78122 | 76215 | 684 | 5.827 | 585 |
| D | alive | 76618 | 71502 | 661 | 6.19 | 582 |
| E | alive | 81862 | 80114 | 631 | 5.895 | 540 |
| F | alive | 93040 | 87678 | 676 | 6.307 | 584 |
| G | alive | 75497 | 73596 | 674 | 6.702 | 602 |
| H | alive | 87218 | 80142 | 651 | 6.679 | 569 |
| I | alive | 80553 | 76464 | 672 | 6.643 | 603 |
| J | alive | 83683 | 80174 | 621 | 6.029 | 531 |
| C0 | h0 | 71840 | 68667 | 286 | 3.431 | 203 |
| D0 | h0 | 53740 | 51722 | 676 | 6.67 | 620 |
| E0 | h0 | 84149 | 80058 | 393 | 4.161 | 303 |
| F0 | h0 | 86070 | 80190 | 396 | 3.814 | 340 |
| G0 | h0 | 86178 | 80125 | 487 | 6.917 | 424 |
| H0 | h0 | 88467 | 80047 | 695 | 7.112 | 606 |
| I0 | h0 | 78143 | 72718 | 556 | 4.987 | 497 |
| J0 | h0 | 82870 | 80278 | 394 | 1.666 | 287 |
| C8 | h8 | 78578 | 77279 | 145 | 1.427 | 79 |
| D8 | h8 | 77824 | 73389 | 613 | 6.451 | 572 |
| E8 | h8 | 84429 | 80104 | 181 | 1.827 | 96 |
| F8 | h8 | 85949 | 80231 | 171 | 2.194 | 97 |
| G8 | h8 | 83537 | 81998 | 167 | 1.539 | 88 |
| H8 | h8 | 75840 | 74195 | 488 | 4.794 | 376 |
| I8 | h8 | 60208 | 58472 | 155 | 1.837 | 111 |
| J8 | h8 | 86459 | 82378 | 190 | 2.094 | 116 |
| C16 | h16 | 99576 | 95611 | 686 | 7.029 | 609 |
| D16 | h16 | 58866 | 56918 | 192 | 2.489 | 142 |
| E16 | h16 | 87631 | 86108 | 140 | 1.421 | 74 |
| F16 | h16 | 56989 | 52437 | 502 | 3.358 | 418 |
| G16 | h16 | 83022 | 80088 | 571 | 3.683 | 487 |
| H16 | h16 | 83141 | 80178 | 527 | 5.344 | 422 |
| I16 | h16 | 82461 | 80120 | 382 | 3.435 | 268 |
| J16 | h16 | 50100 | 46602 | 198 | 2.604 | 149 |
| C.1 | D1 | 85011 | 83211 | 254 | 1.469 | 139 |
| D.1 | D1 | 82449 | 80156 | 378 | 3.358 | 264 |
| E.1 | D1 | 66070 | 64142 | 182 | 1.752 | 117 |
| F.1 | D1 | 54557 | 52615 | 194 | 2.081 | 135 |
| G.1 | D1 | 85937 | 84583 | 148 | 1.443 | 81 |
| H.1 | D1 | 88486 | 80031 | 408 | 3.957 | 284 |
| I.1 | D1 | 86767 | 80113 | 259 | 2.9 | 193 |
| J.1 | D1 | 66293 | 60470 | 72 | 2.356 | 62 |

| | | | | | | |
|---|---|---|---|---|---|---|
| C.3 | D3 | 91301 | 89456 | 361 | 3.417 | 285 |
| D.3 | D3 | 83641 | 80276 | 305 | 3.833 | 247 |
| E.3 | D3 | 66762 | 63676 | 239 | 3.43 | 195 |
| F.3 | D3 | 74009 | 68642 | 83 | 3.025 | 71 |
| G.3 | D3 | 102024 | 92096 | 69 | 2.679 | 45 |
| H.3 | D3 | 86952 | 80109 | 244 | 4.054 | 199 |
| I.3 | D3 | 89236 | 86301 | 274 | 3.44 | 221 |
| J.3 | D3 | 82329 | 80259 | 103 | 2.649 | 77 |
| C.5 | D5 | 92146 | 86179 | 94 | 2.769 | 63 |
| D.5 | D5 | 89258 | 81179 | 112 | 3.052 | 83 |
| E.5 | D5 | 67022 | 65576 | 80 | 3.022 | 66 |
| F.5 | D5 | 83065 | 80114 | 89 | 2.312 | 62 |
| G.5 | D5 | 83399 | 80230 | 69 | 1.786 | 43 |
| H.5 | D5 | 83335 | 80072 | 81 | 1.555 | 57 |
| I.5 | D5 | 82741 | 80249 | 83 | 2.554 | 59 |
| J.5 | D5 | 85826 | 79630 | 84 | 2.447 | 59 |
| C.7 | D7 | 62851 | 61126 | 95 | 3.267 | 77 |
| D.7 | D7 | 83992 | 80238 | 92 | 2.884 | 60 |
| E.7 | D7 | 63148 | 59189 | 74 | 2.641 | 60 |
| F.7 | D7 | 76123 | 68511 | 84 | 3.02 | 64 |
| G.7 | D7 | 85831 | 84604 | 65 | 1.502 | 39 |
| H.7 | D7 | 87489 | 80277 | 118 | 3.177 | 85 |
| I.7 | D7 | 93598 | 89132 | 121 | 2.388 | 83 |
| J.7 | D7 | 83934 | 80218 | 65 | 2.186 | 44 |
| C.9 | D9 | 58473 | 56516 | 78 | 2.444 | 61 |
| D.9 | D9 | 81978 | 80090 | 81 | 2.49 | 56 |
| E.9 | D9 | 73924 | 69046 | 149 | 3.357 | 116 |
| F.9 | D9 | 91913 | 84237 | 170 | 3.55 | 129 |
| G.9 | D9 | 78590 | 74463 | 170 | 2.919 | 132 |
| H.9 | D9 | 84984 | 80148 | 81 | 2.496 | 51 |
| I.9 | D9 | 72000 | 70120 | 75 | 2.45 | 57 |
| J.9 | D9 | 87800 | 82744 | 76 | 1.327 | 52 |
| C.13 | D13 | 85381 | 80131 | 143 | 1.853 | 99 |
| D.13 | D13 | 93840 | 91039 | 89 | 1.173 | 45 |
| E.13 | D13 | 84263 | 80194 | 79 | 2.738 | 49 |
| F.13 | D13 | 66833 | 61172 | 88 | 2.624 | 63 |
| G.13 | D13 | 70792 | 68183 | 207 | 2.979 | 137 |
| H.13 | D13 | 58684 | 53272 | 112 | 2.768 | 86 |
| I.13 | D13 | 81653 | 79822 | 111 | 2.876 | 73 |
| J.13 | D13 | 91436 | 86553 | 393 | 2.096 | 271 |
| C.15 | D15 | 65224 | 60758 | 74 | 2.774 | 67 |
| D.15 | D15 | 85970 | 80149 | 59 | 1.99 | 39 |
| E.15 | D15 | 83338 | 80337 | 55 | 2.458 | 38 |
| F.15 | D15 | 87907 | 80271 | 60 | 1.93 | 43 |

| | | | | | | |
|---|---|---|---|---|---|---|
| G.15 | D15 | 79444 | 71892 | 544 | 5.187 | 438 |
| H.15 | D15 | 73948 | 72426 | 79 | 2.532 | 64 |
| I.15 | D15 | 83923 | 79203 | 126 | 2.477 | 84 |
| J.15 | D15 | 89275 | 83099 | 86 | 1.453 | 54 |

Raw sequences: Unprocessed sequence after sequencing. Clean sequences: Sequence of raw sequences obtained by shearing and filtering. OTU: operational taxonomic unit.

**Supplementary Table S2** Generalized additive models estimating death time utilizing taxonomic phyla, genus and species level indicators from Best Subset Features

| Model | PMI (h) = | Percent (%) | $R^2$(adj.) | GCV |
|---|---|---|---|---|
| | **Phylum** | | | |
| 1 | s(Proteobacteria)+s(Firmicutes)+s(Bacteroidetes)+s(Actinobacteria)+s(unidentified_Bacteria)+s(Spirochaetes)+s(Oxyphotobacteria)+s(Melainabacteria)+s(Fusobacteria)+s(Tenericutes) | 45.0 | 0.344 | 0.792 |
| 2 | s(Proteobacteria)+s(Firmicutes)+s(Bacteroidetes)+s(Actinobacteria)+s(unidentified_Bacteria)+s(Spirochaetes)+s(Oxyphotobacteria)+s (Melainabacteria)+s(Fusobacteria) | 45.0 | 0.354 | 0.769 |
| 3 | s(Proteobacteria)+s(Firmicutes)+s(Bacteroidetes)+s(Actinobacteria)+s(unidentified_Bacteria)+s(Oxyphotobacteria)+s (Melainabacteria)+s(Fusobacteria) | 41.7 | 0.325 | 0.791 |
| 4 | s(Proteobacteria)+s(Firmicutes)+s(Bacteroidetes)+s(Actinobacteria)+s(unidentified_Bacteria)+s(Oxyphotobacteria)+s(*M*elainabacteria) | 39.8 | 0.313 | 0.793 |
| 5 | s(Proteobacteria)+s(Firmicutes)+s(Bacteroidetes)+s(Actinobacteria)+s(unidentified_Bacteria) +s(Melainabacteria) | 39.8 | 0.322 | 0.773 |
| 6 | s(Proteobacteria)+s(Firmicutes)+s(Bacteroidetes)+s(unidentified_Bacteria) +s(Melainabacteria) | 39.8 | 0.331 | 0.752 |
| 7 | s(Proteobacteria)+s(Firmicutes)+s(Bacteroidetes)+s(Melainabacteria) | 39.5 | 0.337 | 0.735 |
| 8 | s(Proteobacteria)+s(Firmicutes)+s(Melainabacteria) | 37.7 | 0.327 | 0.735 |
| 9 | s(Proteobacteria)+s(Firmicutes) | 24.1 | 0.194 | 0.867 |
| 10 | s(Firmicutes) | 16.1 | 0.123 | 0.930 |
| | **Familiy** | | | |
| 1 | s(*Enterococcaceae*)+s(*Enterobacteriaceae*)+s(*unidentified_Clostridiales*)+s(*Corynebacteriaceae*)+s(*Lactobacillaceae*)+s(*Helicobacteraceae*)+s(*Burkholderiaceae*) | 81.4 | 0.757 | 5008.6 |
| 2 | s(*Enterococcaceae*)+s(*Enterobacteriaceae*)+s(*unidentified_Clostridiales*)+s(*Corynebacteriaceae*)+s(*Lactobacillaceae*)+s(*Helicobacteraceae*) | 86 | 0.814 | 3889.2 |
| 3 | s(*Enterococcaceae*)+s(*Enterobacteriaceae*)+s(*unidentified_Clostridiales*)+s(*Corynebacteriaceae*)+s(*Lactobacillaceae*) | 85.6 | 0.809 | 4030.4 |
| 4 | s(*Enterococcaceae*)+s(*Enterobacteriaceae*)+s(*unidentified_Clostridiales*)+s(*Corynebacteriaceae*) | 74.1 | 0.71 | 5130.1 |
| 5 | s(*Enterococcaceae*)+s(*Enterobacteriaceae*)+s(*unidentified_Clostridiales*) | 69.1 | 0.66 | 5938.3 |
| 6 | s(*Enterococcaceae*)+s(*Enterobacteriaceae*) | 62.7 | 0.593 | 7012.5 |
| 7 | s(*Enterococcaceae*) | 46.6 | 0.442 | 9230.9 |

| | | | | |
|---|---|---|---|---|
| | **Genus** | | | |
| 11 | s(*Enterococcus*)+s(*Proteus*)+s(*Lactobacillus*)+s(*unidentified_Clostridiales*)+s(*Vagococcus*)+s(*unidentified_Corynebacteriaceae*)+ s(*unidentified_Enterobacteriaceae*)+s(*Bacteroides*) | 85.3 | 0.792 | 0.307 |
| 12 | s(*Enterococcus*)+s(*Proteus*)+s(*Lactobacillus*)+s(*unidentified_Clostridiales*)+s(*Vagococcus*)+s(*unidentified_Corynebacteriaceae*)+s(*unidentified_Enterobacteriaceae*) | 87.2 | 0.803 | 0.307 |
| 13 | s(*Enterococcus*)+s(*Proteus*)+s(*unidentified_Clostridiales*)+s(*Vagococcus*)+s(*unidentified_Corynebacteriaceae*)+s(*unidentified_Enterobacteriaceae*) | 81.4 | 0.754 | 0.330 |
| 14 | s(*Enterococcus*)+s(*Proteus*)+s(*unidentified_Clostridiales*)+s(*Vagococcus*)+s(*unidentified_Enterobacteriaceae*) | 76.1 | 0.702 | 0.377 |
| 15 | s(*Enterococcus*)+s(*Proteus*)+s(*unidentified_Clostridiales*)+s(*unidentified_Enterobacteriaceae*) | 67.7 | 0.634 | 0.421 |
| 16 | s(*Enterococcus*)+s(*Proteus*)+s(*unidentified_Clostridiales*) | 61.6 | 0.572 | 0.484 |
| 17 | s(*Enterococcus*)+s(*Proteus*) | 44.9 | 0.434 | 0.588 |
| 18 | s(*Proteus*) | 37.4 | 0.338 | 0.708 |
| | **Species** | | | |
| 19 | s(*Enterococcus_faecalis*)+s(*Proteus_mirabilis*)+s(*Clostridium_sporogenes*)+s(*Vagococcus_lutrae*)+s(*Corynebacterium_amycolatum*)+s(*Streptococcus_gallolyticus_subsp_macedonicus*)+s(*Lactobacillus_intestin*alis)+s(*bacterium_mpn_isolate_group_2*)+s(*Lactobacillus_reuteri*)+s(*Falsiporphyromonas_endometrii*) | 59.8 | 0.528 | 0.561 |
| 20 | s(*Enterococcus_faecalis*)+s(*Proteus_mirabilis*)+s(*Clostridium_sporogenes*)+s(*Vagococcus_lutrae*)+s(*Corynebacterium_amycolatum*)+s(*Streptococcus_gallolyticus_subsp_macedonicus*)+s(*Lactobacillus_intestinalis*)+s(*bacterium_mpn_isolate_group_2*)+s(*Lactobacillus_reuteri*) | 59.8 | 0.535 | 0.545 |
| 21 | s(*Enterococcus_faecalis*)+s(*Proteus_mirabilis*)+s(*Clostridium_sporogenes*)+s(*Vagococcus_lutrae*)+s(*Corynebacterium_amycolatum*)+s(*Lactobacillus_intestinalis*)+s(*bacterium_mpn_isolate_group_2*)+s(*Lactobacillus_reuteri*) | 59.8 | 0.541 | 0.530 |
| 22 | s(*Enterococcus_faecalis*)+s(*Proteus_mirabilis*)+s(*Clostridium_sporogenes*)+s(*Vagococcus_lutrae*)+s(*Corynebacterium_amycolatum*)+s(*Lactobacillus_intestinalis*)+s(*bacterium_mpn_isolate_group_2*) | 59.5 | 0.545 | 0.518 |
| 23 | s(*Enterococcus_faecalis*)+s(*Proteus_mirabilis*)+s(*Clostridium_sporogenes*)+s(*Vagococcus_lutrae*)+s(*Lactobacillus_intestinalis*)+ s(*bacterium_mpn_isolate_group_2*) | 59.1 | 0.546 | 0.510 |
| 24 | s(*Enterococcus_faecalis*)+s(*Proteus_mirabilis*)+s(*Clostridium_sporogenes*)+s(*Vagococcus_lutrae*)+s(*Lactobacillus_intestinalis*) | 58.1 | 0.54 | 0.511 |
| 25 | s(*Enterococcus_faecalis*)+s(*Proteus_mirabilis*)+s(*Clostridium_sporogenes*)+s(*Vagococcus_lutrae*) | 56.6 | 0.53 | 0.515 |
| 26 | s(*Enterococcus_faecalis*)+s(*Proteus_mirabilis*)+s(*Vagococcus_lutrae*) | 51.2 | 0.477 | 0.568 |
| 27 | s(*Enterococcus_faecalis*)+s(*Proteus_mirabilis*) | 49.4 | 0.432 | 0.646 |
| 28 | s(*Proteus_mirabilis*) | 25.5 | 0.246 | 0.773 |

The whole models were evaluated by the adjusted $R^2$ value and the generalized cross-validation score (GCV). A higher $R^2$ and lower GCV suggest better model. The percent variation of PMI (%) explained by per model. Created with R (v 3.6.3)[2].

1 Team, R. C. R: A Language and Environment for Statistical Computing, <https://www.R-project.org> (2013).

2 Team, R. C. R: A Language and Environment for Statistical Computing, <https://www.r-project.org/> (2020).
